# An Exceptional Case of Intraparotid Plexiform Neurofibroma Originating from Autonomic Fibers of the Auriculotemporal Nerve

**DOI:** 10.1155/2017/8327215

**Published:** 2017-05-29

**Authors:** Sarantis Blioskas, Sotiris Sotiriou, Katerina Rizou, Triantafyllia Koletsa, Petros Karkos, Anna Kalogera-Fountzila, Konstantinos Markou

**Affiliations:** ^1^1st Department of Otorhinolaryngology, Head and Neck Surgery, Aristotle University of Thessaloniki, AHEPA Hospital, 1 Stilponos Kyriakidi St., 54636 Thessaloniki, Greece; ^2^Pathology Department, Faculty of Medicine, Aristotle University of Thessaloniki, Thessaloniki, Greece; ^3^Department of Radiology, AHEPA Hospital, Aristotle University of Thessaloniki, 1 Stilponos Kyriakidi St., 54636 Thessaloniki, Greece

## Abstract

Plexiform neurofibromas are benign tumors that tend to occur in patients suffering from neurofibromatosis type 1 (NF-1). This report addresses a rare case where the tumor affected the parotid gland, deriving almost exclusively from the peripheral portion of the facial nerve. A 6-year-old male was referred to us complaining about a gradually enlarging swelling over the right parotid area. Imaging localized the lesion to the superficial lobe of the parotid gland, suggesting a neurofibroma. Cosmetic disfigurement and a functional deficit led us to perform complete surgical resection. Meticulous surgical dissection as well as auriculotemporal nerve origin made complete extirpation possible with almost zero morbidity and ensured alleviation of both aesthetic impairment and pain. This is the first case of an intraparotid PN in a pediatric NF-1 patient, which originated from branches of the auriculotemporal nerve and particularly from fibers of the autonomic nervous system. Radical surgical excision was decided according to established decision-making algorithms.

## 1. Introduction

Plexiform neurofibromas (PNs) are benign tumors that usually originate from the nerve sheath of branches of visceral or subcutaneous peripheral nerves. They are often described as rare histological variants of neurofibroma (NF), consisting of a proliferation of cells in the nerve sheath and involving multiple nerve fascicles. Such lesions, which are generally locally invasive, involve/affect a significant length of a major nerve with or without its branches, causing nerve thickness and, in some cases, associated soft tissue hypertrophy. Usually described as a “bag of worms,” PNs are noncircumscribed, baggy, and thick in shape.

Although they can be sporadic, in their vast majority, they tend to occur in patients suffering from neurofibromatosis type 1 (NF-1) [1]. NF-1 results from a spectrum of germline mutations in the NF-1 tumor-suppressor gene which is located in chromosome 17q11 [2]. Patients with this disorder have a higher risk of developing benign and malignant tumors of the Central Nervous System (CNS), peripheral nerve sheath tumors, and other malignant tumors in other parts of the body [1]. Neurofibromas are the most common benign peripheral nerve sheath tumors (PNSTs) that occur in patients with NF-1. They are composed of residuals axons and a mixed population of cells of neoplastic Schwann cells and nonneoplastic fibroblasts, perineural-like cells, and mast cells loosely arranged in an extracellular matrix [2].

The pediatric population is favorably affected since PNs usually grow in early childhood at variable rates. A minority of tumors in the head and neck areas of children affect major salivary glands [1], stemming from either the peripheral portion of the facial nerve within the parotid gland or its terminal branches [3].

In this report we present a rare case of a PN in a 6-year-old male suffering from NF-1. The young male was affected by an intraparotid lesion, which oddly did not derive from the peripheral portion of the facial nerve but instead originated from the auriculotemporal nerve.

## 2. Case Report

A 6-year-old male patient complaining about a growing, painful swelling over the right parotid and submandibular area, was referred to our department for proper evaluation and management. This longitudinal mass had been noticed approximately one year ago. It remained rather small and stable until two months prior to evaluation, at which time it entered a rapid growth phase, nearly doubling in size. As a result, the patient reported mild pain radiating into the periauricular region. Medical and familial history did not reveal significant findings and no previous trauma was reported.

Physical examination included a full head and neck evaluation. Palpation indicated a right-side parotid mass, with a contiguous nodular lesion, approximately 3 cm in diameter. The lesion was painful, noncircumscribed, nonpulsating, rather soft, and of a rubbery consistency while adherent to deep planes. Some smaller nodules appeared at the right postauricular and submandibular region. Due to its size and location, the mass created serious facial disfigurement and asymmetry, with no clinical evidence, however, of any facial weakness or facial nerve impairment. An overall clinical examination also revealed randomly distributed cutaneous café au lait spots on the patient's trunk and extremities (more than 6 and the largest being over 15 mm in diameter). Although these are typical signs of NF-1 we found no other indication of the syndrome such as Lisch nodules or cutaneous neurofibromas.

**Figure 1:**
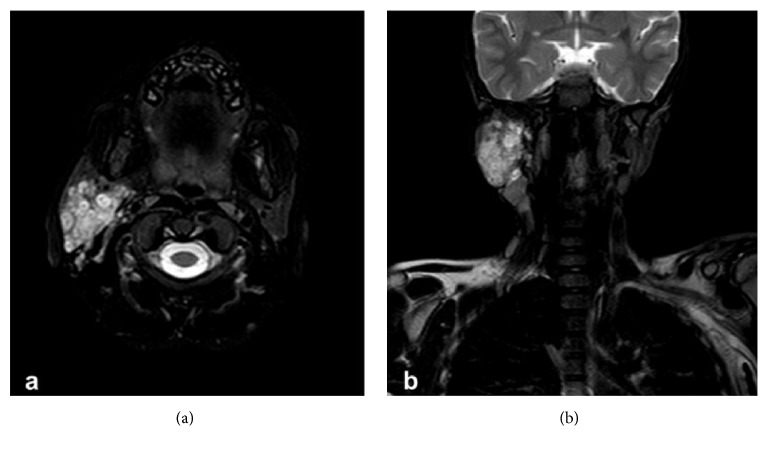
Axial (a) and coronal (b) fat suppressed T2-weighted MR images indicating a large, heterogeneous, lobulated, fusiform, and hyperintense mass in the right parotid region. The multinodular lesion exhibits a target-like appearance due to a peripheral zone of higher intensity. Note the enlarged jugular and spinal accessory lymph nodes.

On these grounds, a gadolinium enhanced magnetic resonance imaging (MRI) was ordered. Imaging confirmed the presence of the mass and localized the lesion to the superficial lobe of the parotid gland, suggesting a neurofibroma adherent to, and possibly originating from, the intraparotid portion of the facial nerve (Figure 1). Fine needle aspiration for cytology (FNA) was performed but was inconclusive.

**Figure 2:**
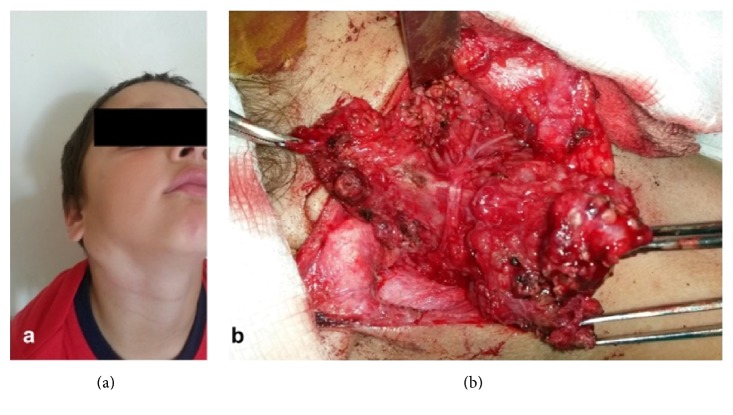
Longitudinal right-side parotid swelling, approximately 3 cm in diameter. The lesion induced serious facial disfigurement and asymmetry (a). Through surgical exploration the lesion was found to be strictly adherent to the main trunk of the facial nerve and longstanding compression had moved the division to temporofacial and cervicofacial branches far more anteriorly than expected. The mass did not, however, originate from the facial nerve, allowing complete resection while preserving neural function (b).

After informed consent was obtained, the patient underwent a typical subtotal superficial parotidectomy, under general endotracheal anesthesia. Intraoperatively, the lesion was found to be a well delimited, tan colored mass, of a gelatine-like consistency which was strictly adherent to the main trunk of the facial nerve. However, accurate tissue-conserving dissection indicated that although in close contact/proximity, it strangely did not originate from the facial nerve. Instead, the neurofibroma seemed to stem from what was identified as the intraparotid branches of the auriculotemporal nerve. Intraoperative view also revealed that longstanding compression to the trunk of the facial nerve had moved the division to temporofacial and cervicofacial branches far more anteriorly than expected, thus hampering dissection (Figure 2). Despite altered anatomy and heavy tumor vascularization, we achieved tumor dissection and excision while ensuring facial nerve integrity.

**Figure 3:**
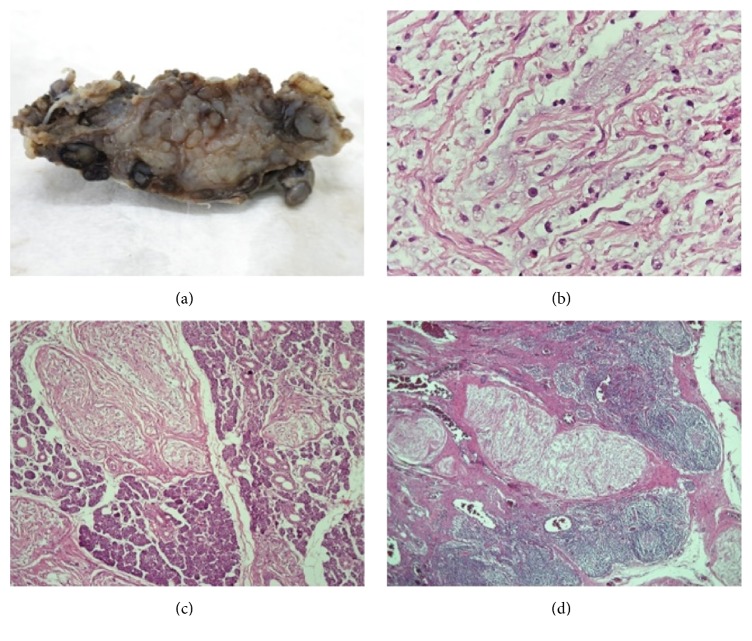
Nodular gray-white soft mass (a) consisting histologically of relatively uniform spindle cells with pointed ends (b), infiltrating the parotid (c), and the hilum of a lymph node (d) ((b) HE ×400; (c) HE ×100; (d) HE ×40).

For histologic examination a nonencapsulated, multilobulated mass measuring 6 × 4.2 × 3 cm and weighting 20 grams was sent to the Pathology Department. Overall, the mass was soft with nodular areas, resembling a “bag of worms.” The cut surface of the mass had a gray-white, glistening appearance, without foci of hemorrhage or necrosis (Figure 3(a)). Hematoxylin and eosin stained sections showed a lesion composed of bundles of nerve fibers infiltrated by Schwann cells, fibroblasts, and perineural-like cells encompassed partly by perineurium. The neoplastic cells were arranged in a fascicular pattern of growth and had a serpentine configuration with elongated nuclei with pointed ends (Figure 3(b)). Neither increased mitotic activity nor atypia were present. The stroma exhibited extensive myxoid degeneration with the presence of strands of collagen and inflammatory aggregates composed of lymphocytes, plasma cells, eosinophils, and scattered mast cells. The neoplasm penetrated the parotic gland thus destructing the normal lobular architecture of the gland (Figure 3(c)). Despite the extensive sampling, no areas of malignant transformation were observed. Interestingly, the neoplasm penetrated an intraparotid lymph node through the hilum followed by blood vessels (Figure 3(d)). The final histopathologic diagnosis was intraneural plexiform neurofibroma.

Overall postoperative course was uneventful and no significant complications were noted, except for a mild transient facial paresis which was fully resolved during postoperative day 1. Cosmetic disfigurement was restored and periauricular pain disappeared. The patient was discharged after 48 hours. Six months later, he remains free of symptoms, postoperative complications, or signs of tumor recurrence.

## 3. Discussion

Intraparotid PNs in paediatric NF-1 patients are extremely rare. To our knowledge there are only 12 reported cases of intraparotid PNs in the English literature, concerning children or adolescents. Out of these, only 9 cases concern NF-1 paediatric patients [3–9]. To add to overall rarity, all of those cases were lesions originating from the trunk or branches of the facial nerve. There is no reference to such tumors originating from different adjacent cranial nerves, let alone the autonomic nervous system.

In our case, the facial nerve was intraoperatively identified to be in close contact with the lesion, yet careful dissection established that it was not the nerve of origin. Instead, the lesion appeared to derive directly from branches of the auriculotemporal nerve. The auriculotemporal nerve arises from the third division of the trigeminal nerve and gives rise to postganglionic parasympathetic fibers travelling from the otic ganglion to the parotid gland. Hence, surgical exploration suggested that autonomic branches of the auriculotemporal nerve were the origin of the lesion. Intraoperative findings were further validated by histological examination. Histology confirmed the extension of the neurofibroma into the hilum of a lymph node. Plexiform neurofibromas can rarely extend into the hilum of lymph nodes and this feature is thought to reflect the diffuse growth of these neoplasms [10]. Yet, since only nerves of the autonomic nervous system can normally be found in lymph nodes, the observed extension into the hilum suggests that plexiform neurofibromas with this feature originate from branches of the autonomic nervous system.

PNs are mainly diagnosed during childhood. Their growth pattern is usually described as gradually progressive, yet rapid growth followed by plateau phases is the norm [11]. Clinical manifestation is majorly influenced by the size and location of the lesions. It is self-evident that neural involvement can cause functional impairment, yet mass effect on adjacent structures (e.g., aerodigestive tract) and cosmetic disfigurement are more common. PNs have traditionally been approached surgically, however, determining the optimal time for surgical intervention continues to raise debate, since recurrence rates are high. Given the invasive nature of the tumor, total excision without significant morbidity remains, in many cases, an unrealistic goal due to functional impingement of the nerve of origin. On the other hand, subtotal resection eventually and almost inevitably leads to tumor regrowth. In addition to residual tumors, Needle et al. [12] pinpointed other prognostic factors correlated with recurrence, such as patient age of less than 10 and tumor location in head and neck subsites. Based on these recommendations, some authors [13] advocate surgery only when significant deformity, potential neural compression, and functional impairment or impending aerodigestive obstruction are in order. More importantly, they stress that even when the above indications are fulfilled, thorough preoperative planning must establish that complete or near complete resection is achievable in the context of preserving neural function, especially where major motor nerves are involved.

In our case, the lesion was first noted at the age of 5, yet it remained subtle and asymptomatic for almost a year, before entering a rapid growth phase. Enlargement resulted in the patient experiencing mild to severe periauricular pain, while excessive tumor size produced serious aesthetical impairment. Cosmetic disfigurement and considerable functional deficit in the form of pain, combined with aggressive growth, induced us to perform surgical resection. Intraoperative discovery of the auriculotemporal nerve origin permitted surgical extirpation with the sole sacrifice of the intraparotid branches of the auriculotemporal nerve, resulting in close to zero morbidity.

Finally it must be mentioned that PNs are relatively common manifestations in NF-1 patients, though not pathognomonic for this syndrome. Diagnosis of NF-1 requires any two of the established seven criteria [14].

In the described case, the presence of plexiform neurofibroma confirmed the diagnosis of NF-1, fulfilling two out of the seven diagnostic criteria (café au lait spots and PN). Early diagnosis of NF-1 in the pediatric population is crucial, since there is an association between von Recklinghausen disease and malignant tumors such as gliomas, leukemias, melanomas, and non-Hodgkin lymphomas [15]. Additionally, when associated with NF-1, PNs have the potential of undergoing malignant sarcomatous transformation, adding considerably to overall morbidity and mortality [15]. Thus, excluding malignancy of an aggressively growing plexiform lesion, the NF-1 diagnosis additionally justified immediate resection.

We present what, to our knowledge, is the first case of a pediatric intraparotid PN of the auriculotemporal nerve in the context of NF-1 to ever be reported. Moreover, we propose that the origin of this neoplasm was the branches of the autonomic nervous system since it penetrated the hilum of a lymph node.

Radical surgical excision undertaken was decided only after vigilant preoperative planning according to rather controversial decision-making algorithms, since high recurrence rates often dictate a “wait and see” policy. Meticulous surgical dissection ensured alleviation of both aesthetic impairment and pain, as well as minimal perioperative morbidity or a postoperative functional deficit.
